# Successful treatment of ptyalism gravidarum with clonidine hydrochloride: A case report

**DOI:** 10.1016/j.crwh.2022.e00409

**Published:** 2022-04-06

**Authors:** Victoria De Braga, Elias M. Dahdouh, Jacques Balayla

**Affiliations:** a Faculty of Medicine, McGill University, 3605 Rue de la Montagne, Montréal, QC H3G 2M1, Canada; b Assisted Reproduction Technology Centre, Department of Obstetrics and Gynecology, CHU Sainte-Justine, Montreal, 3175 Chem. de la Côte-Sainte-Catherine, Montréal, QC H3T 1C5, Canada; c Department of Obstetrics and Gynecology, McGill University, 3605 Rue de la Montagne, Montréal, QC H3G 2M1, Canada

**Keywords:** Ptyalism gravidarum, Clonidine hydrochloride, Sialorrhea, Pregnancy, Case report

## Abstract

Ptyalism gravidarum is a disorder characterized by significant hypersalivation during pregnancy, which affects and interferes with quality of life. No published data has demonstrated an effective approach to treat this condition. This case study reports the use of clonidine hydrochloride, an alpha-2-adrenergic receptor agonist that is typically used as an anti-hypertensive agent, to treat the excessive sialorrhea typical of this disorder. The patient who was treated with this medication saw significant improvement in her symptoms and did not experience any subsequent adverse effects throughout her pregnancy. As a result, we believe that further investigation into this potential treatment for ptyalism gravidarum is necessary ahead of medical guideline incorporation and clinical implementation.

## Highlights

• Clonidine hydrochloride offers a side-effect profile which might aid in treating sialorrhea during gestation.
• Dosages of clonidine hydrochloride up to 0.1 mg daily can be safely given to pregnant women.
• Further studies are needed to evaluate the efficacy and effectiveness of this treatment for ptyalism gravidarum.

## 1 Introduction

Ptyalism gravidarum (PG) is a disorder of unknown origin, defined by excessive salivation affecting pregnant women in early gestation (1). Symptoms include massive saliva volumes (up to 2 l per day), swollen salivary glands, sleep deprivation, significant emotional distress, and social difficulties (2). Patients presenting with this condition are compelled to use cups, tissues, and other measures to dispose of excessive saliva. It usually resolves during the second trimester; however, in some cases, it persists throughout the pregnancy. The pathophysiology of PG is unknown, although beta-human chorionic gonadotropin (b-HCG) and high estrogen levels may be involved. Incidence varies significantly worldwide, from 0.08%, reported in the United States, to up to 0.3%, reported in Japan (1). Associations with hyperemesis gravidarum (HG), male fetal sex, and small for gestational age have been described (3). No definitive treatment for this condition exists ([1], [2], [3]), and the literature has suggested that treatments ranging from hypnosis to dietary and lifestyle changes may help relieve the hyper-salivation. In theory, given that salivary secretion is under neural control via the parasympathetic nervous system, it is hypothesized that a treatment targeting that system might yield success. Several medications such as phenobarbital, piperidolate HCL and alpinia oxyphylla have been tried, without positive results (1). Transdermal scopolamine has been successfully used as a therapeutic option to control drooling in disabled individuals; however, its use to treat hyper-salivation in pregnancy has yet to be elucidated (4). Furthermore, injections of botulinum toxin have emerged as one of the most successful treatments for hyper-salivation, particularly in patients with underlying neurologic disorders (5). However, since the effects of botulinum toxin on a pregnant mother and the fetus have yet to be conclusively elucidated (6), this treatment is not used in pregnancy.

## 2 Case Presentation

A 25-year-old primiparous patient presented at 8 weeks of gestation to the obstetrical clinic for prenatal care. On history, no pertinent medical concerns were elucidated. She reported a history of ovarian cystectomy for a benign ovarian cyst five years prior. Other than prenatal vitamins, she reported no medication use or history of allergies. Her gynaecological history was otherwise unremarkable. She was of normal height and weight and reported no family history of adverse obstetrical outcomes. Ultrasound examination confirmed the presence of a singleton, live intrauterine pregnancy whose dates corresponded to menstrual age. Non-invasive prenatal screening (NIPS) revealed a male fetus with a low risk of aneuploidy. First-trimester prenatal labs were likewise reported as unremarkable.

Prenatal follow-up continued normally until 15 weeks of gestation, when the patient began experiencing hyper-salivation, with volumes of sialorrhea reaching over a litre daily (>1350 cc daily (DIE)). She reported significant social and professional impairments and difficulty remaining asleep given the chronic sensation of fluid in the oral cavity. The hyper-salivation rendered fluid and solid intake almost impossible. Though she had persistent nausea in the first trimester, unlike most cases of ptyalism gravidarum, no diagnosis of hyperemesis gravidarum had been made. Dietary and lifestyle modifications (ingestion of foods rich in absorbent starch, frequent small meals, dry crackers, fluid restriction, gum intake and sucking on ice) did not alleviate the symptoms.

By 18 weeks of gestation, given her normal vital signs and persistence of symptoms, a low-dose (0.05 mg–0.1 mg DIE PRN) trial of the anti-hypertensive, alpha-2 agonist clonidine hydrochloride was attempted – which successfully treated her condition and restored quality of life. She reported only mild headaches, which were short-lived and did not interfere with daily activities. The patient went on to take the clonidine hydrochloride as needed for the remainder of her pregnancy whenever the severity of the hyper-salivation could not be tolerated. Her blood pressure remained stable throughout the second and third trimesters, demonstrating an average decrease of 10 mmHg in the systolic pressure and 8 mmHg in the diastolic. The pregnancy concluded with an overall maternal weight gain of 16 lbs., and the delivery of a healthy male neonate at 39 weeks of gestation, with normal weight at 3100 g, Apgar scores of 9–9 at 1 and 5 min respectively, and normal umbilical cord pH parameters.

## 3 Discussion

Clonidine hydrochloride is a medication used to treat hypertension. As an alpha-2-adrenergic receptor agonist, clonidine acts to stimulate alpha receptors within the medulla oblongata and hypothalamus, effectively disrupting sympathetic transmission to the heart, kidneys, and peripheral vasculature while simultaneously increasing vagal tone (7,8). Vagal tone is a representation of the activity of the vagus nerve, a fundamental component of the parasympathetic branch of the autonomic nervous system (9). Since alpha-2 receptors are widely distributed within the body, the influence of an alpha-2-adrenergic agonist can be vast. Clinical effects of clonidine hydrochloride include reductions in blood pressure, heart rate, total peripheral resistance, plasma renin concentrations, and urinary aldosterone and catecholamines. Its most common dose-dependent side-effect (occurring in about 40 of 100 people), however, is xerostomia, or dry mouth (7,10). Although highly inconvenient for people prescribed this medication for hypertension, this unwelcome side-effect can be of use to people suffering from hyper-salivation. The use of clonidine hydrochloride during pregnancy has previously been investigated by Rothberger et al. (11). They concluded that while this medication has been shown to reduce maternal blood pressure by approximately 9 mmHg, it may impact fetal growth when higher doses (>0.15 mg/day) are administered. However, if this medication is the only antihypertensive given, its total dose is limited to 0.05–0.10 mg DIE, and maternal heart rate response is regularly monitored, a regimen can be established to manage maternal blood pressure without compromising potential fetal growth. In the case of this patient, the target endpoint would be to prescribe clonidine hydrochloride for its side-effect profile of reducing hyper-salivation and not to control blood pressure, but the effects on the growing fetus remain. In addition, researchers at the Université de Montréal have conducted a case series to describe the efficacy of clonidine hydrochloride for pregnant patients with ptyalism gravidarum, in particular, within their hospital, in addition to reviewing the literature on the use of this medication during pregnancy (12). They concluded that more data is necessary to adequately describe the population who might benefit from this treatment for hyper-salivation. Therefore, given the proven safety and use of this medication in pregnancy, the patient described in this case study was counselled accordingly, and she accepted a trial of this medication which alleviated her symptoms and restored quality of life.

Based on the current literature, ptyalism gravidarum is not considered to be a “serious” condition requiring medical treatment. However, the social and interpersonal inconvenience necessitates satisfactory management for this rare complication. In addition, there has yet to be a report of successful medical treatment of this condition. In this case, clonidine hydrochloride, an antihypertensive medication proven to cross the placental barrier yet be safe during all trimesters of pregnancy (8,13), was selected to be used off-label to exploit its main side-effect (dry mouth), ultimately to treat the extreme hyper-salivation of pregnancy experienced by this patient. The off-label use of drugs in pregnancy and reproduction is not uncommon nor discouraged (14,15); therefore, it would not be inconceivable for clonidine hydrochloride to have an analogous off-label benefit, capable of treating hyper-salivation during gestation. Furthermore, since this medication has been proven to be a safe antihypertensive agent in pregnancy (8), it can be reasoned that titrated dosages can be safely administered to normotensive women without fear of significant blood pressure alterations or adverse effects. Further studies evaluating the advantage of this medication in pregnant women suffering from ptyalism gravidarum are necessary prior to widespread clinical implementation.
